# VIRTUAL ONLINE COMMUNITIES FOR AGING LIFE EXPERIENCE (VOCALE) APPROACH: PILOT STUDIES

**DOI:** 10.1093/geroni/igz038.3174

**Published:** 2019-11-08

**Authors:** Oleg Zaslavsky, Annie T Chen, Andrew Teng, Shih-Yin Lin, Soojeong Han, Frances Chu, George Demiris

**Affiliations:** 1 University of Washington, Seattle, Washington, United States; 2 NYU Rory Meyers College of Nursing, New York, New York, United States; 3 University of Pennsylvania School of Nursing, Philadelphia, Pennsylvania, United States

## Abstract

Emerging evidence suggests behavioral strategies focusing on symptom management can reduce frailty symptoms and improve quality of life. Unfortunately, these interventions are rarely scalable for implementation in geriatric care. Contemporary online technologies have tremendous potential for addressing this need. We developed and pilot tested an approach entitled Virtual Online Community for Aging Life Experience (VOCALE). The approach had two stages. In the first stage, we piloted the use of a Facebook platform to engage older adults with frailty symptoms in ten-week moderated discussions on health-related topics. In the second study, we used data from stage one to develop a prototypical persona of a person with frailty symptoms. The persona was then incorporated into an eight-week Facebook intervention informed by problem solving therapy to facilitate self-management in another group of older adults with frailty symptoms. The results from both rounds showed that it was feasible to recruit, engage, and retain persons ages 69-92 into virtual online community interventions. Attrition ranged from 25% to 33% in rounds one and two. In both rounds, we observed positive trends of change in health measures such as general health self-efficacy, disease self-efficacy, and health literacy. Throughout the studies, older adults shared multiple posts concerning their experience with age-related symptoms and described their self-management practices. These projects, which leveraged a common social media platform, demonstrated preliminary efficacy of an online intervention for frailty management. If confirmed, this approach might provide a viable model for other medically complex geriatric conditions where self-management is essential.

